# World Health Organization (WHO) surgical safety checklist implementation and its impact on perioperative morbidity and mortality in an academic medical center in Chile

**DOI:** 10.1097/MD.0000000000003844

**Published:** 2016-06-10

**Authors:** Hector J. Lacassie, Constanza Ferdinand, Sergio Guzmán, Lorena Camus, Ghislaine C. Echevarria

**Affiliations:** a División de Anestesiología, Facultad de Medicina, Pontificia Universidad Católica de Chile, Santiago, Chile; b Clínica Las Condes, Santiago, Chile; c División de Cirugía, Facultad de Medicina, Pontificia Universidad Católica de Chile, Santiago, Chile; d Facultad de Medicina, Pontificia Universidad Católica de Chile, Santiago, Chile; e Department of Anesthesiology, Perioperative Care & Pain Medicine, New York University School of Medicine, New York, NY.

**Keywords:** checklist, Latin America, morbidity, mortality, surgery

## Abstract

Health care organizations are unsafe. Numerous centers have incorporated the WHO Surgical Safety Checklist in their processes with good results; however, only limited information is available about its effectiveness in Latin America. We aimed to evaluate the impact of the checklist implementation on the in-hospital morbidity and mortality rate in a tertiary health care center. After Institutional review board approval, and using data from our hospital administrative records, we conducted a retrospective analysis of all surgical encounters (n = 70,639) over the period from January 2005 to December 2012. Propensity scoring (PS) methods (matching and inverse weighting) were used to compare the pre and postintervention period, after controlling for selection bias. After PS matching (n = 29,250 matched pairs), the in-hospital mortality rate was 0.82% [95% confidence interval (CI), 0.73–0.92] before and 0.65% (95% CI, 0.57–0.74) after checklist implementation [odds ratio (OR) 0.73; 95% CI, 0.61–0.89]. The median length of stay was 3 days [interquartile range (IQR), 1–5] and 2 days (IQR, 1–4) for the pre and postchecklist period, respectively (*P* < 0.01).

This is the first Latin American study reporting a decrease in mortality after the implementation of the WHO Surgical Checklist in adult surgical patients. This is a strong and simple tool to make health care safer, especially in developing countries.

## 1 Introduction

Since the publication of “To Err is Human.”^[1]^ worldwide awareness of medical error has driven the need to control it in the best possible ways. Health care organizations are unsafe, reporting adverse events ranging between 3% and 17%, accounting for up to 98,000 deaths/year due to medical errors.^[1]^ Surgical procedures are responsible for 1 adverse event every 300 of them.^[2]^

There is substantial evidence that almost one half of complications or adverse events derived from surgical procedures are preventable. Other risk industries (military, nuclear power plants, aviation, etc.) have developed ways to avoid mistakes by implementing teamwork, improving communication skills, and other strategies to deal with their inherited hazards.

In 2008, the World Health Organization (WHO) and their study group “Safe Surgery Saves Lives” published a document recommending the use of a surgical checklist to decrease the risk of preventable accidents during surgical procedures. They demonstrated a decrease in major complications from 11% to 7% and reduced mortality in 53% (from 1.5% to 0.8%).^[3]^ Since then, the WHO Surgical Safety Checklist has been implemented in more than 4100 hospitals and in 1790 of them is being used actively.^[4]^ The use of the WHO surgical checklist has been associated with reduced complications and mortality rates, better adherence to safety standards, improved communication and teamwork, and economic benefits.^[5]^

In Latin America, some countries have endorsed the use of the WHO Surgical Safety Checklist; however, there is paucity on their results after implementation. Our aim is to determine the impact of the implementation of the WHO Surgical Safety Checklist in terms of morbidity and mortality in adult surgical patients in a tertiary health care institution in Chile.

## 2 Methods

After Institutional review board approval (Facultad de Medicina, Pontificia Universidad Católica de Chile, Santiago, Chile), we conducted a retrospective analysis of all surgical encounters, including cardiothoracic surgeries, on patients age 15 years and above from January 2005 to December 2012 at a large urban academic medical center. All encounters before September 2009 were classified as “no checklist performed,” and after as “checklist performed,” respectively. All procedures performed outside the operating room, except for those performed in the labor and delivery room, were excluded.

Before September 2009, the policy regarding safety in surgery did not include the use of a formal surgical checklist and safety measures were not systematically assessed.

For the adoption of the Surgical Checklist, we used recommended strategies for implementation that included recruitment of local experts in key surgical areas, such as surgeons, anesthesiologists, operating room head nurses, and surgical nurse coordinators; language adaptation of the WHO surgical checklist; operating room personnel training through mock procedures; multimedia material demonstrating the correct use of the surgical checklist; and easy access to a copy of the WHO surgical checklist in every operating room.

The administrative database analyzed, stripped of all patient identifiers, was provided by our hospital. Each encounter included up to 14 diagnostic and procedure *International Classification of Diseases, 9th Revision, Clinical Modification* (ICD-9-CM) codes, demographic data, date of admission and discharge, emergency status, health care system used, and in-hospital death. A 5-level “high-risk” variable was created in order to account for surgical complexity and associated in-hospital mortality (level 1, surgeries with <1% in-hospital mortality; level 2, 1% to <5%; level 3, 5% to <10%; level 4, 10% to <15%; level 5, > or =15%).^[6]^

Surgical heterogeneity was calculated by the Internal Herfindahl Index, which represents the diversity or comprehensiveness of the types of procedures performed at a facility.^[7]^

### 2.1 Statistics

We used propensity score (PS) analysis to control for differences in baseline characteristics. The PS is the conditional probability of receiving an exposure (e.g., checklist) given a set of measured covariates. To estimate the PS, a logistic regression model was used in which “treatment” status (checklist performed vs not performed) was regressed on the baseline (pre-treatment) characteristics (Table 1).

**Table 1:**
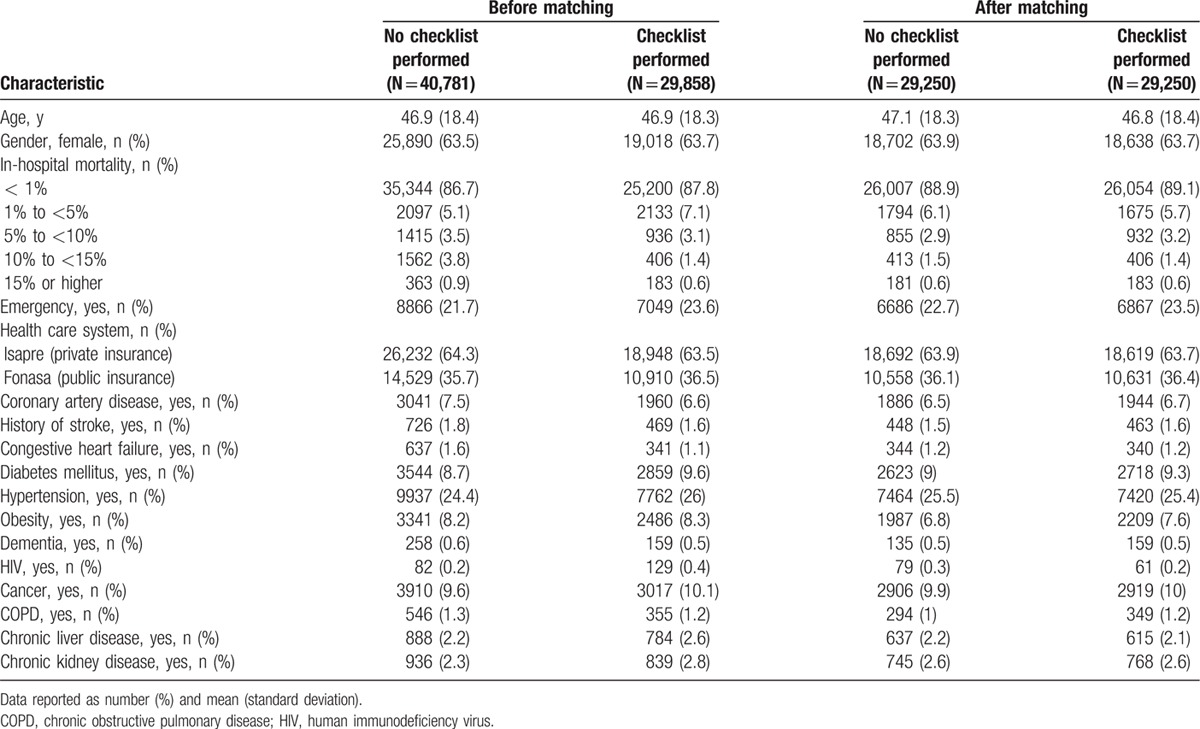
Patient characteristics.

PS analysis was implemented in 2 ways to control for confounding:^[8]^

1. PS matching: we performed the matching using a one-to-one nearest neighbor caliper matching without replacement with a caliper size of 0.2 standard deviations. Balances in the distribution of baseline covariates were assessed by estimating absolute standardized differences of the covariates between the 2 groups before and after matching. Any imbalanced covariates (standardized difference >10%) after matching were adjusted for in the final analysis.^[9]^ As the PS-matched sample does not consist of independent observations, we used a marginal regression model with robust standard errors.
2. PS weighting: we weighted the entire sample by the inverse probability of the treatment weights derived from the PS. If a subject has a higher probability of being in a group, it is considered over-represented and therefore is assigned a lower weight. Conversely, if the subject has a smaller probability, it is considered as under-represented and is assigned a higher weight. We then fit a weighted linear regression model using an indicator variable representing checklist intervention status as the sole predictor, and mortality as our outcome variable.

Data are expressed as mean (SD; standard deviation) or median (IQR; interquartile range) unless otherwise stated. A 2-sided *P* value less than 0.05 was considered significant. The analyses were performed using STATA/SE v.12.0 (StataCorp LP, College Station, TX).

## 3 Results

A total of 70,639 encounters were identified during the studied time frame. Of these, in 29,858 cases, a checklist was performed (42.3%). The baseline characteristics of the patients are listed in Table 1. The Internal Herfindahl index was 0.12 (SD 0.01).

**Figure 1:**
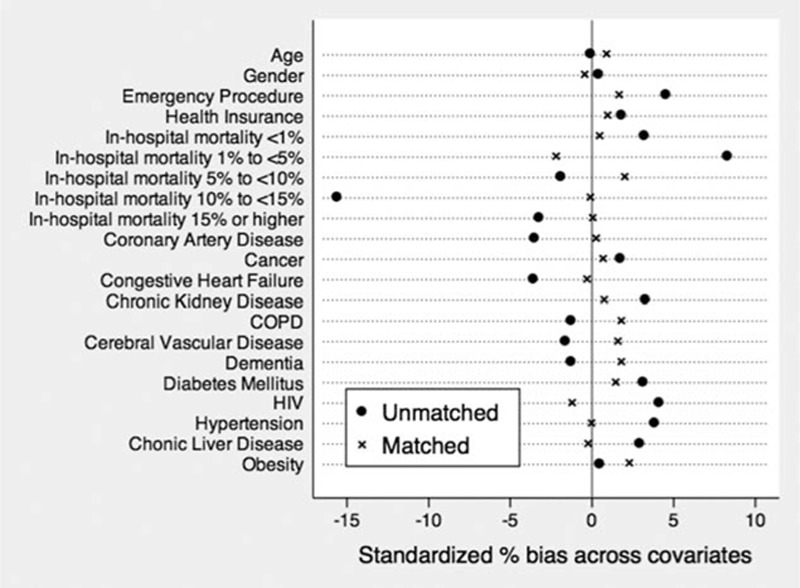
Absolute standardized differences in baseline covariates between pre and postchecklist before and after propensity score matching (postmatch standardized difference <10% indicates covariate balance). COPD, chronic obstructive pulmonary disease; HIV, human immunodeficiency virus.

We were able to match 29,250 patients from the checklist group (98%) with an excellent balance in the covariates (Fig. 1).

The results of the logistic regression model for the primary outcome—in-hospital mortality—are summarized in Table 2. After PS matching, the in-hospital mortality rate was 0.79% [95% confidence interval (CI), 0.69–0.89] before and 0.61% (95% CI, 0.46–0.71) after checklist implementation [odds ratio (OR) 0.73; 95% CI, 0.61–0.89]. Similar results were obtained using PS weighting, based on the average treatment effect on the treated (OR 0.79; 95% CI, 0.66–0.95).

**Table 2:**
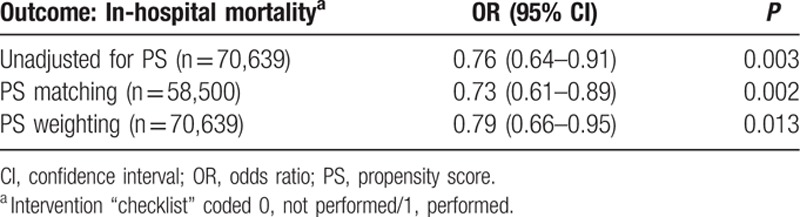
Propensity score (PS) analysis: comparison of in-hospital mortality of patients in whom a checklist was and was not performed

The mean length of stay (LOS) in the matched sample was 3 (IQR 1–5) days and 2 (IQR 1–4) days for the pre and postchecklist period, respectively (*P* < 0.01). No significant differences in the odds of postoperative surgical site infection^i^ were obtained between time periods (estimated OR 1.13, 95% CI, 0.94–1.37).

## 4 Discussion

To our knowledge, this is the first Latin American report on the effectiveness of the WHO Surgical Safety Checklist. We were able to find a decrease in mortality as well as a decrease in the length of hospital stay, after the implementation of the checklist in adult surgical patients.

In September 2009, the administrative authorities decided to incorporate the WHO Surgical Safety Checklist as a standard of care at our institution, generating an opportunity to conduct a quasi-experimental study design by comparing the population before and after the intervention.^[10]^

This study was performed in a tertiary 500-bed, high complexity hospital where more than 12,000 surgeries are made every year, with an Internal Herfindahl Index of 0.12, representing a high heterogeneity in surgical procedures, with a standard of care comparable to other high complexity centers in developed countries. Recently, the implementation of the surgical safety checklists in Ontario, Canada, was not associated with significant reductions in operative mortality or complications.^[11]^ It is plausible to think that the checklist may unmask institutions that have not reached yet high quality standards of care. In our case, despite having high standards for our country, we think that there is still room for improvement in medical care, as it was demonstrated by having almost 30% less odds of dying by applying the checklist.

The WHO Surgical Safety Checklist use has gone beyond the operating rooms, where it was originally intended to be used. de Vries et al^[12]^ prospectively applied a multidisciplinary checklist that follows the surgical pathway from admission to discharge in 8 Dutch high standard of care hospitals, demonstrating a decrease in complications and mortality. Our study showed a decrease in mortality and LOS, without applying further checklist besides the intraoperative one. We speculate that we may have found larger differences should we have pursued a more comprehensive checklist. For the sake of simplicity and to achieve more compliance with the performance of the lists, we opted for this strategy.

In the seminal study by Haynes et al,^[3]^ they incorporated 8 hospitals from every continent, except from Latin America. Our report acknowledges that the WHO Surgical Safety Checklist is a valid instrument for reducing morbidity and mortality in the Latin American population as well. We thought that our standard of care was high enough not to find differences by the implementation of the checklist, but we were wrong. We speculate that its use in hospitals in a region with lesser quality standards will have a higher impact on clinical outcomes.

Adapting to the use of a procedural checklist represents a cultural change that may be initially uncomfortable for participants.^[13]^ The WHO has recommended some strategies to increase the likelihood of success upon implementation of the surgical checklist, which include early engagement of staff; active leadership and identification of local champions; extensive discussion, education and training; multidisciplinary involvement; coaching; ongoing feedback; and local adaptation.^[14]^ We embraced most of the suggestions. They are time-consuming and depend upon champions in each group involved, as well as a motivated group of people that fortunately we had. We think that the effort is worthwhile and should be adopted universally. Successful system change requires demonstrating the need for change, engaging institutional leadership, collecting data, and most important, providing training in teamwork so that everyone feels respected and accountable.^[15]^ Qualitative analysis suggested that the effectiveness hinges on the ability of implementation leaders to persuasively explain why and adaptively show how to use the checklist.^[16]^ We followed this suggestion and prepared champions and leaders for implementation. It seems that the strategy worked considering our results. Besides, it has persisted in time and the use of the checklist is ubiquitous throughout the operating rooms.

In a recent study, a list of 227 high-risk operations in patients 65 years and older was identified. It allowed them to disclose a difference in inpatient mortality for high-risk procedures performed on patients 65 years and older double the pooled inpatient mortality for the procedures on this list with a mortality of at least 1% for patients younger than 65 years (6% vs 3%).^[6]^ We incorporated these ICD-9-CM codes in our PS model, to control for those high-risk surgeries known to affect postoperative mortality.

Semel et al^[17]^ performed a cost analysis for the hospital comparing implementation of the checklist to existing practice in U.S. hospitals. Hospital cost savings are relatively insensitive to variation in implementation in a hospital with a baseline major complication rate after surgery of at least 3%.^[17]^ In our case, the baseline complication rate was not possible to obtain from the administrative records; however, the decrease in LOS and mortality might have had a real economic impact. Hospital administrators should strongly support the use of the WHO surgical checklist.

Several limitations regarding our study are worth noting. Because of the nonrandomized nature of the study, the checklist use was influenced by an administrative decision. Consequently, differences in outcomes among patients in whom we did and did not use the checklist may be explained by confounding. Also, surgeries and techniques were not homogenous and the information regarding postoperative care was not available in the database. We attempted to address this issue by implementing a PS analysis to balance the treatment groups and controlling for all measured covariates, in particular the surgical complexity and associated in-hospital mortality, linked to the patients’ prognosis. Second, we report a slight lesser mortality incidence than other reports. This might be due to the design of the current study by reporting in-hospital mortality and not the 30-day occurrence. Maybe our approach is stricter and makes the results more relevant. Third, we did not evaluate compliance nor completeness and thoroughness of the application of the checklist. Fourth, we used administrative data to assess surgical complications. Although this method is commonly used, it is inferior to prospective measurement or chart review.^[11]^ Finally, there may be concerns about the perception of patients when doing the checklist before anesthesia. Today, we know that patients give strong support to the use of surgical checklists, which is a powerful challenge to those physicians resistant to its use.^[18]^ Besides, when patients are aware of the checks being performed, this does not provoke anxiety.^[19]^ Therefore, from the patient's point of view, there are no significant barriers for performing the ritual.

In summary, the WHO Surgical Safety Checklist is a strong and simple tool to make health care safer, especially in developing countries.

## Footnotes

i ICD-9-CM codes 998.5, 998.51, 998.59, and 674.3x were used.
